# Spinal Adhesive Arachnoiditis: A Literature Review

**DOI:** 10.7759/cureus.33697

**Published:** 2023-01-12

**Authors:** Jadhe Maillard, Sávio Batista, Felipe Medeiros, Gabriela Farid, Paulo Eduardo Santa Maria, Caio M Perret, Stefan W Koester, Raphael Bertani

**Affiliations:** 1 Faculty of Medicine, Estácio de Sá University, Rio de Janeiro, BRA; 2 Faculty of Medicine, Federal University of Rio de Janeiro, Rio de Janeiro, BRA; 3 Serviço De Neurocirurgia, Hospital Municipal Miguel Couto, Rio de Janeiro, BRA; 4 Neurological Surgery, Hospital Municipal Miguel Couto, Rio de Janeiro, BRA; 5 Neuroscience, Federal University of Rio de Janeiro, Rio de Janeiro, BRA; 6 Neurological Surgery, Vanderbilt University School of Medicine, Nashville, USA; 7 Neurosurgery, Hospital Municipal Miguel Couto, Rio de Janeiro, BRA

**Keywords:** spinal arachnoiditis, postoperative, obstetric epidural, obstetric complication, arachnoiditis, anesthesia, adhesive arachnoiditis

## Abstract

Adhesive arachnoiditis (AA) is a rare inflammatory and scar-forming disease with several etiologies that may lead to incapacitating sequelae if not managed early. Nevertheless, as the onset of symptoms varies from days to years, the etiology is not often discovered. The disease is characterized by adhesions disrupting the cerebrospinal fluid flow and causing encapsulation and atrophy of the nerve root. Therefore, a range of clinical features may be present, including urinary, gastroenterology, dermatologic, and neurologic. In terms of diagnosis, magnetic resonance imaging is the gold standard showing pseudocysts with adherent and narrow nerve roots toward the center of the dural sac or peripherally cluster and narrow nerve roots with empty thecal sac. Despite its sensitivity and specificity, the imaging findings are not often associated with clinical manifestations, requiring treatment being based on anamneses and clinical findings. Nowadays, AA can be managed with pharmacological and non-pharmacological treatment, although none provides a completely satisfying result.

## Introduction and background

Adhesive arachnoiditis (AA) is a debilitating condition characterized by persistent arachnoid inflammation leading to intrathecal scars and dural adhesions [1-3]. Although being considered a rare disease, the exact incidence is unknown and probably underestimated due to the omission of subclinical cases [4].

Several conditions may lead to AA, including mechanical, chemical, infectious, and previous spinal illness [2,5,6]. Despite the low incidence of AA, the increasing rates of back surgeries, the most common etiology nowadays, enhance the frequency of arachnoiditis [7]. Thereby, the knowledge of clinical and imaging characteristics is essential to achieve early diagnosis and prevent unwanted complications [8].

Although some people are asymptomatic, others present with variable neurological deficits that without early diagnosis and proper management may progress to an incapacitating condition [9,10]. Moreover, similar symptoms to more common diseases such as disc herniation, multiple sclerosis, and spinal cord tumors [2,11,12] can delay the treatment and lead to devastating sequelae [8].

This paper provides a review of AA describing the mechanism, clinical features, diagnosis, and treatment to increase knowledge and alertness of this condition, allowing early detection and intervention to attenuate sequels of this incurable disease.

Methodology

Study Design and Identification

This is a literature review based on the following guiding question: “What are the most relevant scientific pieces of evidence concerning adhesive arachnoiditis?” The study was performed using PubMed, Embase, Web of Science, ScienceDirect, Cochrane, and Scopus using the following keyword: “adhesive arachnoiditis.” This keyword was searched in the modality “all fields.”

Eligibility Criteria

Letters to the editor, commentaries, and articles that were not strongly related to AA were excluded from the review.

Studies Selection

Two independent reviewers (J.M. and S.B.) performed the data analysis and extraction, with disagreements settled by a senior author (R.B.). Initially, the search strategy revealed a total of 2,294 articles, of which 790 were excluded by duplications, and 1,439 by title and abstract. After reading the remaining 65 full papers, 31 were selected to make part of this review.

## Review

Etiology

Several etiologies have been described in the literature, such as infection, including tuberculosis [5,13], syphilis [5,13], brucellosis [14], candida [13], mycosis [14], and HIV infection [2]; mechanical causes, including spinal surgery [15], trauma [8], and disc herniation [8]; previous spinal illness, including ankylosing spondylitis [5,6], autoimmune vasculitis [2], and Guillain-Barré syndrome [2]; and chemical causes, including morphine [16], myelograms with oil-based radiographic contrast agents [17,18], sulfite-containing preservatives [2], phenolic solutions [10], chlorhexidine [19], epidural injection of steroids and antibiotics [5], blood in subarachnoid hemorrhage [20,21] or epidural blood patch [22], and epidural anesthetics [14,23]. Nevertheless, epidural analgesia in obstetrics using preservative-free, low-concentration bupivacaine with opioids is still not established as a defined cause for AA [1].

Moreover, a familial case of chronic adhesive spinal arachnoiditis has been reported, suggesting that AA may have a genetic autosomal dominant trait [24].

Although the specific cause is not often determined since the onset of symptoms varies from days to years [9,25], the site and etiology of AA may be associated. Formerly, the predominant locations of AA were cervical and thoracic spinal segments, with infections, mainly tuberculosis, being the principal cause [7]. Nowadays, the main incidence of AA is in the lumbar spinal segment due to an increase in the number of lumbar spine procedures [12].

Pathology

The inflammation process and progression that occurs in AA was first described by Charles Burton in 1970 [1]. Idiopathic or with a known etiology, the process is based on injury and inflammation of the pia-arachnoid [25], followed by granulomatous tissue formation, fibrosis, and adhesions [5]. However, the genetic predisposition to aberrant fibrinolytic scarring pathways in the role of AA is still uncertain [25].

The inflammation results in nerve root edema and hyperemia, leading to fibrinous exudate and radicular symptoms [1,25]. Cerebrospinal fluid (CSF) carries and dilutes phagocytes and fibrinolytic enzymes, forming fibrinous bands [25,26] and starting the adhesive phase. In this step, the cytokines carried by the CSF added to the avascular nature of the arachnoid layer compromise the healing process [27]. Collagen deposition in the fibrinous bands forming scars and causing ischemia [15], encapsulation, and atrophy of nerve roots and the cord [14] are some characteristics of this phase. Afterward, the scar formed disrupts CSF circulation, diminishing nutrients to neural components [25] and leading to debilitating pain and neurological deficits [14]. Moreover, the impairment of CSF flow may result in syrinx formation by creating a pressure gradient toward the obstructed area, with an interstitial leakage into the spinal cord [26].

The nerve roots and vascular compression may be responsible for the acute neurologic decline. However, the progression of the ischemia and scar formation proceed to neurologic damage, which can be present as back pain and neurological deficits over weeks to months [15].

In addition, the chronic inflammation present in late-stage AA can lead to intrathecal bony metaplasia resulting in arachnoiditis ossificans [26]. Spinal cord swelling, myelomalacia, cauda equina syndrome, and hydrocephalus are other possible complications [2,27,28].

Clinical presentation

The AA ranges from the onset of symptoms and levels of severity, which may vary from eight days to 17 years, and subclinical to incapacitating neurologic symptoms, respectively [12]. Despite most cases being asymptomatic and coincidental findings [26], the symptomatic ones often present as a chronic, painful, and debilitating condition [2] and frequently are not associated with magnetic resonance imaging (MRI) findings, not justifying the clinical presentation [7]. Besides the extensive array of clinical features and their intensity, the symptoms can also be static or progressive during the course of the disease [7].

The AA manifestation is often related to the site of injury, and as lumbar surgeries are the most common cause of AA, back pain is the main complaint. Beyond having the characteristic of worsening with activity, restriction of mobility in the lumbosacral spine [10], decreased truncal range of motion [1], burning pain in the lower back that may radiate down the legs [8], and back spasms [8] are other possible findings related to back pain.

Symptoms in the lower limbs may be present and manifest as paresthesia in the extremities [1,2], spams [8], myeloradiculopathy [2], weakness [1,12] paralysis [8], impaired touch and vibration sense [12], loss of thermal sensation [7], pathological reflexes such as Lasegue's sign [10,24], pain typical of the sciatic nerve distribution, difficulties in controlling limbs if an efferent motor nerve root is affected [2], gait imbalance [2], leg pain usually bilateral [1], burning ankles and feet [8], and partial or complete paralysis of the lower extremities [8].

Bowel and urinary manifestations including neurogenic bowel and bladder [2], constipation [3], urinary sphincter dysfunction [1] leading to urinary retention or incontinence, and urinary urgency [3,10,24] are often present in the late course of the disease [2]. Moreover, other findings such as headache [29], vertigo [29], sexual dysfunction [2], anorexia [1], cryptogenic skin rash, and itching [8] can also be seen.

Diagnosis

The diagnosis is based on thorough anamnesis, clinical presentation, and imaging evaluation [30]. The site of injury may be associated with clinical and imaging findings, and as the lumbar is the most affected area, the characteristic triad represented by back pain, neurological deficits, and MRI representing adhesions is often present in symptomatic patients [7]. Nevertheless, MRI findings do not often relate to the clinical presentation of the AA, so the management of the disease must be made by the clinical exam and history, and not according to the severity of the disorder presented on imaging [7,26].

Analyzing the clinical presentation, the lumbar spine is the most affected spinal site due to the higher frequency of low back surgery [7]. Moreover, low back pain is often associated with weakness of lower limbs, sensory changes, and strength with differing degrees of reflex compromise [2,10]. Furthermore, as those symptoms are not pathognomonic of AA, other spinal pathologies must be considered as differential diagnoses, such as disc herniation, multiple sclerosis, spinal cord tumors, postlaminectomy pain syndrome, and neurosarcoidosis [2,11,12].

In terms of imaging, myelography was used to diagnose AA; however, the intrathecal iodinated contrast given was precipitating the disorder [12]. Nowadays, MRI is the gold standard, having 92% sensitivity and 100% specificity [27,30], and is usually performed with contrast to elucidate inflammation [7]. In MRI, AA may be presented as pseudocysts with adherent and narrow nerve roots toward the center of the dural sac or peripherally cluster and narrow nerve roots with empty thecal sac [6,30]. The cysts may also show as uni or multilocular with septa [29]. Furthermore, soft tissue replacement in subarachnoid space and adhesions impairing cerebral spinal fluid resulting in a partial or complete obstruction may also be present [7,25].

In post-subarachnoid hemorrhage patients with persistent headaches and myelopathic changes, MRI is indicated to exclude arachnoiditis [21]. In cases of negative MRI, the thecaloscopy may assist diagnose loculated arachnoid cysts presented in arachnoiditis [2]. Furthermore, to exclude the presence of tumors when cord swelling and intramedullary increased signals are present, a spinal cord biopsy is indicated [2].

Treatment

AA management is based on early pharmacological or surgical intervention, which may be associated with physical therapy to improve spinal fluid flow and minimize scarring [2,27]. Although there is no specific protocol management, the main objective of treatment is to avoid permanent damage by suppressing neuroinflammation and promoting neuroregeneration and pain control, improving the patient's quality of life [2]. Nevertheless, most severe cases can be refractory to aggressive treatment, remaining lasting disability [25]. In patients with acute efficacy of treatment, the follow-up of AA is mandatory as the disease may recur after 20 years of treatment [20].

Frequently, the treatment is initiated with pharmacologic management [27]. Medical remedies such as nonsteroidal anti-inflammatory drugs [2,12] and pulse steroid therapy, most commonly methylprednisolone [8,10,12], have been described to treat inflammation, although only a few studies show its effectiveness in preventing scar tissue [30]. In cases of pain, the pharmacologic therapy may include naltrexone, gabapentin [1,3,18], pregabalin [2,12], tricyclic antidepressants [2,12], muscle relaxants such as baclofen [2], local anesthetics [12], opioid analgesics [2,10], non-opioids [10], and narcotics [8]. Furthermore, although epidural injections improve pain, their use is not recommended, as it may exacerbate AA [2].

In terms of surgical techniques, several procedures are described in the invasive management of AA, demonstrating the lack of a specific and effective method [12,27]. In general, the surgical treatment pursues to release the cicatricial adhesions and repair CSF flow, albeit its efficacy is uncertain, as surgery increases the inflammatory process and is scar-forming [27]. Therefore, in the short term, surgery may relieve symptoms; however, in the long term, it is inefficient [26,30].

The flexible endoscopy is a minimally invasive technique demonstrated to be efficient, safe, and superior to the microscope providing better visualization of the arachnoid adhesions [20]. Thecaloscopy, arachnoidolysis, neural stimulation, subarachnoid shunting, cyst fenestration, myelotomy, duraplasty, and laminectomy are some other described techniques [2,10,30]. In the short term, arachnoid dissection and subarachnoid space decompression appear to be efficient, although, in long-term evolution, there are no satisfactory results [25,31].

In addition, to improve clinical prognosis by untethering the spinal cord and recovering CSF flow, microdissection of thickened adherent arachnoid followed by ventricle-subarachnoid shunt may be used [31].

Neural mobilization, a non-pharmacological and non-surgical treatment, relieves symptoms and enhances the quality of life by disrupting adhesion by manual therapy or exercise. This mechanism reduces intraneural edema and restores homeostasis to enhance nerve function. Although studies showed that the technique may be effective, randomized controlled trials are required to determine long-term efficacy [27].

Furthermore, the incidence of depression and suicide in AA disease is high due to the lasting disabilities associated with psychosocial burden, requiring multimodal and interprofessional pain management [14,20].

## Conclusions

AA is an uncommon inflammatory disease predominantly located in the lumbar spine with an extensive array of presentations. This disorder is characterized by inflammation, scar-forming, cluster and narrow nerve roots, progressing to neurologic deterioration. Several causes such as infections, multiple back surgeries, and neurodegenerative disorders were described, although the etiology is not always clear, as the disease may appear in days, months, or years after the responsible event.

Clinical presentations vary from subclinical to a range of symptoms, such as urinary, gastroenterology, dermatologic, and neurologic manifestations, which may lead to incapacitating sequelae. Nevertheless, early perception and management of AA are essential to prevent unfavorable outcomes such as symptoms progression and irreparable damages. The diagnosis of AA is based on the history, clinical, and MRI features, including pseudocysts with adherent and narrow nerve roots, although MRI imaging is not often associated with clinical presentations. In addition, different pharmacological and surgical treatments have been reported focusing primarily on pain relief, symptom control, and life quality improvement, although there is no gold-standard management with a guarantee of success. In some cases, it may also be necessary to associate physical therapy, cognitive-behavioral therapy, and other forms of psychotherapy with a better and more holistic result.
